# Comparing the variances of several treatments with that of a control treatment: Theory and applications

**DOI:** 10.1371/journal.pone.0296376

**Published:** 2024-01-12

**Authors:** Jingsen Kong, Hezhi Lu

**Affiliations:** 1 School of Economics, Jinan University, Guangzhou, China; 2 School of Economics and Statistics, Guangzhou University, Guangzhou, China; 3 Lingnan Research Academy of Statistical Science, Guangzhou University, Guangzhou, China; LUMSA: Libera Universita Maria Santissima Assunta, ITALY

## Abstract

A common and important problem in medicine, economics and environmental studies is the comparison of the variances of several treatments with that of a control treatment. Among the existing methods, Spurrier’s optimal test based on multivariate *F* distribution has exact type I error rates. However, it requires equal sample sizes among the treatment groups. To extend the application scope, in this paper, we propose a new efficient test for comparing several variances with a control using the marginal inferential model (MIM). Simulation studies show that the MIM test guarantees the exact type I error rate whether the sample size is equal or unequal. Moreover, the power of the MIM test is competitive with that of Spurrier’s optimal test. Finally, two real examples are used to demonstrate the application of the proposed method.

## Introduction

In medicine, economics and environmental studies, there are many situations wherein *k* independent populations are compared to a control population with respect to scale parameters [1]. For example, in medicine studies, the variability of testosterone levels in men in various groups classified according to their smoking habits is compared to the variability of testosterone levels in healthy people. [2] indicated that smoking has a negative impact on testosterone levels, leading to less variability, as some of them may have low testosterone levels for other reasons, while healthy persons will have high levels. In this situation, a common problem is whether different types of smokers, e.g., former smokers, light smokers or heavy smokers, have less testosterone variability than nonsmokers. Thus, in the case of *k* treatment populations *π*_1_, *π*_2_, …, *π*_*k*_ and a control population *π*_0_, where the observations from the *i*th population follow the normal distribution $N\left(\mu_{i},\sigma_{i}^{2}\right),\;i=0,\;1,\ldots ,\;k$, the interest is to test the hypothesis

$$H_{0}:\sigma_{i}^{2}=\sigma_{0}^{2},\forall \;i\;vs\;H_{1}:\sigma_{i}^{2}\leq \sigma_{0}^{2},\;i=1,\ldots ,\;k\;with\;at\;least\;one\;strict\;inequality,$$ (1)

where $\sigma_{i}^{2},\;i=0,\;1,\ldots ,\;k$ is the variance of the *i*th population.

Many studies focus on multiple comparisons of treatments with a control or standard with respect to a parameter of interest under order restrictions. [3, 4] proposed a standard multiple comparison procedure for comparing several treatments with a control. [5] constructed confidence intervals for comparing several normal variances with a control variance in multifactor experiments. [6] provided an algorithm for constructing multiple hypothesis testing. To improve the mean half-square successive difference statistic [7, 8] proposed a modified percentile bootstrap method for comparing the variances of two independent groups. Moreover, a combination of Levene-type tests with a finite-intersection method for testing the equality of variances against ordered alternatives can be found in [9]. [10] discussed the quality of F-ratio resampling tests for comparing variances. Note that all the test methods mentioned above for comparing variances have been found to be unsatisfactory in terms of type I error probabilities or powers.

To obtain an optimal test procedure that has exact control behavior in the type I error rate [11, 12], recommended the use of sample quasi ranges as a measure of variance. The distribution of quasi ranges in samples from a normal population was discussed by [13]. [14] classified normal populations with respect to a control using sample quasi ranges on censored data. [15] discussed optimal designs for comparing several experimental treatment variances with that of a standard treatment variance. A one-sided test based on sample quasirange was proposed by [16] to test homogeneity against a simple ordered alternative. [17] proposed a test based on isotonic estimators for testing the equality of variances of several normal populations against tree-ordered alternatives. Moreover [1], proposed an upper one-sided test based on the sample quasi range to test the homogeneity of variances from the normal population with that of the control population. By computing exact critical constants, the sample quasirange method can control the type I error rate at a preassigned level, *α*.

The sample quasirange approach aims at provably efficient inference, and the corresponding test can guarantee the exact type I error rate. Moreover, different from other test methods, Spurrier’s optimal test [15] is a single step test procedure, while other test methods based on sample quasi ranges can be regarded as a step-up test procedure for multiple hypothesis testing. Moreover, some studies consider testing whether the variances of *k* populations are not equal to the variance of a control population. However, under certain circumstances, one-sided simultaneous confidence intervals provide more inferential sensitivity than two-sided simultaneous confidence intervals [18]. For example, some upper one-sided tests for comparing several normal variances with a control variance can be found in [1, 15, 17–19].

For lower one-sided tests [15], provided the optimal test procedure for hypothesis test (1). More specifically, suppose that *n*_1_ = *n*, *n*_1_ = *n*_2_ = … = *n*_*k*_ = *m*, denote the sample variance for treatment by $S_{i}^{2},i=0,1,2,\ldots ,k$. Define the random variables

$$F_{i}=\frac{S_{i}^{2}/\sigma_{i}^{2}}{S_{0}^{2}/\sigma_{0}^{2}},i=1,\ldots ,k,$$ (2)

and the test statistics

$$W_{i}=\frac{S_{i}^{2}}{S_{0}^{2}},i=1,\ldots ,k.$$ (3)

The distribution of (*F*_1_, …, *F*_*k*_) is a multivariate *F* distribution and the marginal distribution of *F*_*i*_ is the *F* distribution with *m* − 1 and *n* − 1 degrees of freedom, *i* = 1,2, …, *k*. Letting *F*_(1)_ = min⁡(*F*_1_, …, *F*_*k*_), the *p*-value of the Spurrier test is given by

$$p=1-P\left(F_{\left(1\right)}>c\right)=1-\int_{0}^{\infty }{\left\{1-H\left[\frac{xc\left(m-1\right)}{n-1}\right]\right\}}^{k}g\left(x\right)dx,$$ (4)

where $c=min\{s_{1}^{2}/s_{0}^{2},s_{2}^{2}/s_{0}^{2},\ldots ,s_{k}^{2}/s_{0}^{2}\}$ is the ratio of minimal sample variance among the *k* treatments and the sample variance with the control, *H* is the cdf of χ^2^ (*m* − 1) and *g* is the pdf of χ^2^ (*n* − 1). In general, (4) can be well approximated using the Gauss-Laguerre numerical quadrature using subroutines to evaluate *H*.

Spurrier’s test [15] has greater applicability while ensuring competitive efficiency, but it requires equal sample sizes among the treatment groups. [19] indicated that one may design an experiment that meets the sample size requirement, but the final available data might be unequal as a result of unexpected losses. Hence, the goal of this paper is to construct a more efficient test for general cases involving equal and unequal sample sizes based on inferential model [20] theory.

## Methodology

### Marginal inferential model framework

Different from frequentist and Bayesian inference methods, Fisher and Dempster intended to propose prior-free inference frameworks that produce probabilistic inferential results with desirable frequency properties. However, the small sample properties for the fiducial argument [21, 22] and Dempster-Shafer theory [23, 24] may not be calibrated for meaningful probabilistic inference. As an alternative [20], proposed an inferential model (IM) framework for prior-free probabilistic inference. In fact, IM has some connections to fiducial inference and Dempster-Shafer theory. The key difference between these three methods is the way to work out for auxiliary variables. In particular, the IM’s handling of the auxiliary variables can guarantee desirable frequency properties for all sample sizes.

In marginal inference problems, where only parts of the full parameter are of interest [25], developed a marginal inferential model (MIM) framework for marginal inference. The key idea of the MIM is to reduce the dimension of the auxiliary variable. In general, MIM starts with a system of equations, called association, representing a statistical model with unknown parameter *θ* = (*ψ*,*ξ*) for observable data *X* ~ *P*_*X*|*θ*_ via an auxiliary random variable *U*. The initial model is expressed as if the goal were to simulate, i.e.,

$$p\left(X,\theta \right)=a\left(U,\theta \right),\;U\sim P_{U},$$ (5)

where *p* and *a* are known functions and *U* has a known distribution function. To emphasize that *θ* = (*ψ*,*ξ*), we can rewrite the association as

$$p\left(X;\psi ,\xi \right)=a\left(U;\psi ,\xi \right),\;U\sim P_{U}.$$ (6)

Note that IM consists of a three-step inference procedure, which includes an association step (A-Step), a prediction step (P-Step), and a combination step (C-Step). Since *ψ* is the parameter of interest and *ξ* is the nuisance parameter, the marginal IM has the following three steps:

**A-Step**: Suppose that there are functions *q*, *b* and *c*, and new variables *V* = (V_1_,V_2_) ~ *P*_*V*_, such that (6) can equivalently be written as

$$q\left(X,\psi \right)=b\left(V_{1},\psi \right),$$ (7a)

$$c\left(X,V_{2},\psi ,\xi \right)=0.$$ (7b)

Since the exact value of *V*_2_ does not provide any information about the interest parameter *ψ*, there is no benefit to retaining component (7b) and trying to predict the auxiliary variable *V*_2_. Clearly, the key idea of the MIM is that *V*_1_ is generally of a lower dimension than *U*. Therefore, the MIM for *ψ* is only based on the following association:

$$q\left(X,\psi \right)=b\left(V_{1},\psi \right),\;V_{1}\sim P_{V_{1}}.$$ (8)

The dimension-reduction step of the MIM guarantees efficient inference properties. The result of A-Step is a set-value mapping given by

$$\Theta_{X}\left(V_{1}\right)=\{\psi :\;X=b\left(V_{1},\psi \right)\}.$$ (9)

**P-Step**: Following [7], the auxiliary variable *V*_1_ can be predicted by specifying an optimal predictive random set $S\left(V_{1}\right)\sim P_{S(V_{1})}$, that is,

$$P_{S(V_{1})}\{P\left\{V_{1}\in S\left(V_{1}\right)\right\}\geq 1-\alpha \}\leq \alpha .$$ (10)

If $V_{1}^{*}$ is a real value for the unobserved *V*_1_, from credible conditions [7], then *S*(*V*_1_) should be even better in the sense that there is a high probability that $V_{1}^{*}\in S(V_{1})$.

**C-Step**: Combine the association and predictive random set *S*(*V*_1_) to obtain the final predictive random subset of *ψ*,

$$\Theta_{X}\left(S\left(V_{1}\right)\right)=\underset{V_{1}\in S\left(V_{1}\right)}{⋃}\Theta_{X}\left(V_{1}\right).$$ (11)

According to the inference framework of IM, given an assertion of interest *A*, MIM also provides two probabilistic measure functions about the uncertainty of *A*. The belief function (*bel*_*X*_ (*A*)) and plausibility function (*pl*_*X*_ (*A*)) are

$${bel}_{X}\left(A\right)=P\left(\Theta_{X}\left(S(V_{1})\right)\subseteq A\right),$$ (12)

$${pl}_{X}\left(A\right)=1-{bel}_{X}\left(A^{∁}\right)=P\left(\Theta_{X}\left(S(V_{1})\right)⊈A^{∁}\right).$$ (13)

In fact, *bel*_*X*_ (*A*) and *pl*_*X*_ (*A*) can be regarded as the minimum and maximum probabilities that support the truth of assertion *A*. Note that the plausibility function can be easily used to create a frequentist decision rule. We can reject the null hypothesis if *pl*_*X*_ (*A*) ≤ *α*. Moreover, the MIM 100(1 − *α*)% confidence interval can be obtained by computing confidence limits from {*ψ*: *pl*_*X*_ (*ψ*) > *α*}.

The IM-based method is exact in the sense that it does not need any asymptotic approximation. Moreover, IM’s output has a meaningful interpretation within and not just across experiments. According to [20], the IM test is valid if

$$\underset{\theta \in A}{\text{sup}}P_{X|\theta }\left\{{pl}_{X}\left(A\right)\leq \alpha \right\}\leq \alpha \;for\;all\;\alpha \in \left(0,1\right).$$ (14)

Moreover, if “≤ *α*" can be replaced by “= *α*", then the MIM method is efficient.

### The proposed MIM test

In this section, we propose a marginal IM-based method for testing hypothesis (1). Suppose that the observations $X_{1}^{i},\;X_{2}^{i},\;\ldots ,\;X_{n_{i}}^{i}$ from the *i*th population *π*_*i*_ follow the normal distribution $N\left(\mu_{i},\sigma_{i}^{2}\right)$, the null hypothesis in (1) can be transferred to an assertion $B=\{\sigma_{i}^{2}=\sigma_{0}^{2},\;\forall \;i\}$, where *i* = 0,1, …, *k*, *n*_*i*_ ∈ {1, 2,3, …, *l*}, *k* and *l* are positive integers. If evidence from the observable data suggests that the assertion *B* is false, the null hypothesis would be rejected. Let ${\bar{X}}_{i}=\sum_{j=1}^{n_{i}}X_{j}^{i}/n_{i}$ and $S_{i}^{2}={\sum_{j=1}^{n_{i}}(X_{j}^{i}-{\bar{X}}_{i})}^{2}/(n_{i}-1)$. According to [26], the conditional association model based on the minimal sufficient statistics for $\left(\mu_{i},\sigma_{i}^{2}\right)$ is given by

$${\bar{X}}_{i}=\mu_{i}+{\sigma_{i}n}_{i}^{-1/2}U_{i}\;and\;\left(n_{i}-1\right)S_{i}^{2}=\sigma_{i}^{2}V_{i}^{2},\;i=0,\;1,\;\ldots ,k,$$ (15)

where $U_{i}\sim N\left(0,1\right),\;V_{i}^{2}\sim \chi^{2}\left(n_{i}-1\right)$, *U*_*i*_ and $V_{i}^{2}\;\left(i=0,\;1,\;\ldots ,k\right)\;$ are independent. Moreover, this association model can be equivalently simplified as

$${\bar{X}}_{i}=\mu_{i}+S_{i}n_{i}^{-1/2}U_{i}/V_{i}\;and\;\left(n_{i}-1\right)S_{i}^{2}=\sigma_{i}^{2}V_{i}^{2},\;i=0,\;1,\;\ldots ,k.$$ (16)

Here $\sigma_{i}^{2}$ are parameters of interest. For any ${\bar{X}}_{i},\;S_{i},\;\sigma_{i}^{2}$ and *U*_*i*_/*V*_*i*_, there exist a *μ*_*i*_ such that ${\bar{X}}_{i}=\mu_{i}+S_{i}n_{i}^{-1/2}U_{i}/V_{i},\;i=0,\;1,\;\ldots ,k$. Since there is no direct information that can be obtained about $\sigma_{i}^{2}$ by knowing *U*_*i*_/*V*_*i*_, there is no benefit to retain the first equation in (16). To reduce the dimension of the auxiliary variable, we can ignore *U*_*i*_ (*i* = 0, 1, …, *k*) and work with auxiliary variables *V*_*i*_ (*i* = 0, 1, …, *k*) directly. Therefore, the initial association model of the marginal IM can be expressed as

$$\frac{\left(n_{i}-1\right)S_{i}^{2}}{\sigma_{i}^{2}}=V_{i}^{2},\;V_{i}^{2}\sim \chi^{2}\left(n_{i}-1\right),\;i=0,\;1,\ldots ,k.$$ (17)

Note that the associations (17) play two distinct roles. Before experiment, the associations characterize how likely the observable $S_{i}^{2},\;i=1,\ldots ,k$ to be. Once $S_{i}^{2},\;i=1,\ldots ,k$ are observed, the true parameter $\sigma_{i}^{2},\;i=1,\ldots ,k\;$ can be obtained by solving the above equations. Clearly, the true value of $V_{i}^{2},\;i=1,\;\ldots ,\;k$ will never be known, but we know exactly the distribution of $V_{i}^{2},\;i=1,\;\ldots ,\;k$. Therefore, we can predict the $V_{i}^{2},\;i=1,\;\ldots ,\;k$, so that we can make inference about $\sigma_{i}^{2},\;i=1,\ldots ,k$.

However, the difficulty we encountered are as follows: On one hand, since the unknown parameters is (*k* + 1)-dimentional, so the auxiliary random variable should also be (*k* + 1)-dimentional, which might lead to poor efficiency especially when *k* is large. On the other hand, the hypothesis actually has constraints, i.e.,

$$\Theta =\left\{\left(\sigma_{0}^{2},\sigma_{1}^{2},\ldots ,\sigma_{k}^{2}\right):\sigma_{1}^{2}\leq \sigma_{0}^{2},\sigma_{2}^{2}\leq \sigma_{0}^{2},\ldots ,\sigma_{k}^{2}\leq \sigma_{0}^{2},\right\}.$$ (18)

Since the parameters have constraints, it is challenging to make inferences about the assertion *B*. Some techniques or strategies are needed.

First, we take a different perspective about (18), i.e., (18) is regarded as the parameter space rather than a constraint. This is reasonable when we look at the null hypothesis and alternative hypothesis in (1). If we take (18) as the parameter space, a straightforward transformation of (1) is

$$H_{0}:\text{min}\left\{\sigma_{1}^{2},\sigma_{2}^{2},\ldots ,\sigma_{k}^{2}\right\}=\sigma_{0}^{2}\;vs\;H_{1}:\text{min}\left\{\sigma_{1}^{2},\sigma_{2}^{2},\ldots ,\sigma_{k}^{2}\right\}<\sigma_{0}^{2}.$$ (19)

Define

$$\theta =\frac{\text{min}\left\{\sigma_{1}^{2},\sigma_{2}^{2},\ldots ,\sigma_{k}^{2}\right\}}{\sigma_{0}^{2}}.$$ (20)

Then the hypothesis can be transferred to

$$H_{0}:\theta =1\;vs\;H_{1}:\theta <1.$$ (21)

We can see that (21) and (1) are equivalent under (18). Therefore, we only need to infer about assertion *B* = {*θ* = 1}. Note that assertion *B* only contains one-dimensional parameters. Next, we rewrite association (17) as follows:

$$\frac{S_{0}^{2}}{\sigma_{0}^{2}}=\frac{V_{0}^{2}}{\left(n_{0}-1\right)},$$ (22)

$$\frac{S_{i}^{2}}{\sigma_{i}^{2}}=\frac{V_{i}^{2}}{\left(n_{i}-1\right)},\;i=1,\ldots ,k.$$ (23)

Using partial information ($\sigma_{1}^{2}=\sigma_{2}^{2}=\ldots =\sigma_{k}^{2}$) from the null hypothesis in (1), $\text{min}\left\{\sigma_{1}^{2},\sigma_{2}^{2},\ldots ,\sigma_{k}^{2}\right\}$ is a constant. Hence, the minimum of $s_{i}^{2}$ is corresponds to the minimum of $V_{i}^{2}/\left(n_{i}-1\right),\;i\in \{1,\;2,\;\ldots ,\;k\}$, i.e.,

$$\frac{\text{min}\left\{S_{1}^{2},S_{2}^{2},\ldots ,S_{k}^{2}\right\}}{\text{min}\left\{\sigma_{1}^{2},\sigma_{2}^{2},\ldots ,\sigma_{k}^{2}\right\}}=\text{min}\left\{\frac{V_{1}^{2}}{\left(n_{1}-1\right)},\frac{V_{2}^{2}}{\left(n_{2}-1\right)},\ldots ,\frac{V_{k}^{2}}{\left(n_{k}-1\right)}\right\},$$ (24)

Combining equations (22) and (24), (20) can be written as

$$\frac{S_{0}^{2}}{\text{min}\left\{S_{1}^{2},S_{2}^{2},\ldots ,S_{k}^{2}\right\}}\theta =\frac{\frac{V_{0}^{2}}{\left(n_{0}-1\right)}}{\text{min}\left\{\frac{V_{1}^{2}}{\left(n_{1}-1\right)},\frac{V_{2}^{2}}{\left(n_{2}-1\right)},\ldots ,\frac{V_{k}^{2}}{\left(n_{k}-1\right)}\right\}}.$$ (25)

Clearly, the auxiliary variable $\frac{V_{0}^{2}}{\left(n_{0}-1\right)}/\text{min}\left\{\frac{V_{1}^{2}}{\left(n_{1}-1\right)},\frac{V_{2}^{2}}{\left(n_{2}-1\right)},\ldots ,\frac{V_{k}^{2}}{\left(n_{k}-1\right)}\right\}$ can be separated from data $S_{0}^{2}/\text{min}\left\{S_{1}^{2},S_{2}^{2},\ldots ,S_{k}^{2}\right\}$. According to [27], the distribution of $\frac{V_{0}^{2}}{\left(n_{0}-1\right)}/\text{min}\left\{\frac{V_{1}^{2}}{\left(n_{1}-1\right)},\frac{V_{2}^{2}}{\left(n_{2}-1\right)},\ldots ,\frac{V_{k}^{2}}{\left(n_{k}-1\right)}\right\}$ does not rely on the observable data $\left\{{S_{0}^{2},S}_{1}^{2},\ldots ,S_{k}^{2}\right\}$. Let *F* denote the distribution function of the right side of equation (25), and we can construct a new MIM-based procedure to make inferences about assertion *B* = {*θ* = 1} as follows:

**A-Step**: The final association model of the MIM is given by

$$\Theta_{X}\left(u\right)=\left\{\theta :\;\frac{S_{0}^{2}}{\text{min}\left\{S_{1}^{2},S_{2}^{2},\ldots ,S_{k}^{2}\right\}}\theta =F^{-1}\left(u\right)\right\},$$ (26)

where *u*~*Unif*(0,1) and *F*^−1^ is the inverse function of *F*.

**P-Step**: Different kinds of assertions have different expression forms of the corresponding valid predictive random sets. For a left-sided assertion (21), a possible optimal predictive random set *S*(*u*) for predicting the auxiliary variable *u*, that is,

$$S(u)=\left\{u:0\leq u\leq U\right\},U\sim Unif\left(0,\;1\right).$$ (27)

**Theorem 1.** According to [20], for a left-sided assertion, the predictive random set *S*(*u*) = {*u*: 0 ≤ *u* ≤ *U*},*U*~*Unif*(0, 1) is optimal in the sense that *P*_*S*(*u*)_ {*u* ∈ *S*(*u*)} ~ *Unif*(0,1).

**Proof.** Let $Q_{S\left(u\right)}\left(u\right)=P_{S(u)}\left\{u\notin S\left(u\right)\right\}=1-U,U\sim Unif\left(0,\;1\right)$. The predictive random set *S*(*u*) is optimal in the sense that *P*_*U*_{*Q*_*S*(*u*)_ (*U*) ≥ 1 − *α*} = *α* for each *α* ∈ (0,1); hence

$$P_{S\left(u\right)}\left\{u\in S\left(u\right)\right\}\sim Unif\left(0,1\right).$$

Hence, the proof is complete.

**C-Step**: Combine (26) and (27), we have

$$\Theta_{X}\left(S(u)\right)=\left\{\theta :U\geq F\left(\frac{S_{0}^{2}}{\text{min}\left\{S_{1}^{2},S_{2}^{2},\ldots ,S_{k}^{2}\right\}}\theta \right)\right\}=\left\{\theta :\frac{S_{0}^{2}}{\text{min}\left\{S_{1}^{2},S_{2}^{2},\ldots ,S_{k}^{2}\right\}}\theta \leq F^{-1}\left(U\right)\right\}.$$ (28)

Then, the plausibility function for assertion *B* = {*θ* = 1} is

$$\begin{array}{ll} pl\left(B\right) & =P\text{(}\Theta_{X}\left(S\left(u\right)\right)\cap B\neq \emptyset \}=1-P\left(U\leq F\left(\frac{S_{0}^{2}}{\text{min}\left\{S_{1}^{2},S_{2}^{2},\ldots ,S_{k}^{2}\right\}}\right)\right)\\ & =1-F\left(\frac{S_{0}^{2}}{\text{min}\left\{S_{1}^{2},S_{2}^{2},\ldots ,S_{k}^{2}\right\}}\right). \end{array}$$ (29)

**Theorem 2.** The proposed MIM inference method can control the type I error rate at a preset level *α* ∈ (0,1), i.e.,

$$\underset{\theta \in B}{\text{sup}}P_{X|\theta }\left\{pl\left(B\right)\leq \alpha \right\}=\alpha \;for\;all\;\alpha \in \left(0,1\right).$$

**Proof.** Since $F\left(\frac{S_{0}^{2}}{\text{min}\left\{S_{1}^{2},S_{2}^{2},\ldots ,S_{k}^{2}\right\}}\theta \right)=u\sim Unif\left(0,1\right),\;$ and

$$\begin{array}{ll} \underset{\theta \in B}{\text{sup}}\;P_{X|\theta }\left\{pl\left(B\right)\leq \alpha \right\} & =\underset{\theta \in B}{\text{sup}}\;P_{X|\theta }\left\{1-F\left(\frac{S_{0}^{2}}{\text{min}\left\{S_{1}^{2},S_{2}^{2},\ldots ,S_{k}^{2}\right\}}\theta \right)\leq \alpha \right\}\\ & =\underset{\theta \in B}{\text{sup}}\;P_{X|\theta }\left\{F\left(\frac{S_{0}^{2}}{\text{min}\left\{S_{1}^{2},S_{2}^{2},\ldots ,S_{k}^{2}\right\}}\theta \right)\geq 1-\alpha \right\}=\alpha . \end{array}$$

Hence, the proof is complete.

## Simulation study

The proposed marginal IM method is an accurate testing method, and the efficiency of MIM inference does not require simulation verification. However, the *p* value of Spurrier’s optimal test needs to be approximated using the Gauss-Laguerre numerical quadrature. In fact, it is an approximate method. Moreover, due to the asymptotic properties of large samples, the performance of the two test methods tends to be consistent. For better comparison, we conduct Monte Carlo simulations to assess the performances of the MIM-based test and Spurrier’s test in various small sample situations. The parameters of the comparison are mainly the type I error rate and power. The parameter settings refer to [20] and [10]. In the experiment, we consider various cases with different sample sizes (*n*_0_, *n*_1_, …, *n*_*k*_) and different treatment groups $\left(\sigma_{0}^{2},\sigma_{1}^{2},\ldots ,\;\sigma_{k}^{2}\right)$ for *k* = 3, 5, and 7. In each case, we repeat the experiment 10,000 times to evaluate the significance level of 5% which provides a 95% confidence interval of the type I error rate as (0.0457, 0.0543) for a 5% error rate. Tables 1 to 3 summarize the results of the comparisons of the type I error rates and empirical powers of these two methods for small sample sizes. Note that the first component of the combination (*n*_0_, *n*_1_, …, *n*_*k*_) is for the control group; for example, (*n*_0_, *n*_1_, *n*_2_, *n*_3_) = (5, 6, 7, 8) represents that the control group has five samples.

**Table 1:** The estimated type I error rates and powers for *k* = 3.

	$\left(\sigma_{0}^{2},\sigma_{1}^{2},\;\sigma_{2}^{2},\;\sigma_{3}^{2}\right)$					
	(2, 2, 2, 2)		(2, 2, 2, 1)		(2, 1, 1, 1)	
(*n*_0_, *n*_1_, *n*_2_, *n*_3_)	MIM	Spurrier	MIM	Spurrier	MIM	Spurrier
5, 5, 5, 5	0.0493	0.0494	0.0834	0.0838	0.1504	0.1498
15, 15, 15, 15	0.0473	0.0473	0.2039	0.2041	0.3910	0.3909
30, 30, 30, 30	0.0483	0.0482	0.4039	0.4030	0.6431	0.6439
5, 10, 10, 10	0.0458	0.0457	0.1212	0.1214	0.2361	0.2358
10, 20, 20, 20	0.0488	0.0493	0.2179	0.2188	0.3728	0.3737
20, 30, 30, 30	0.0493	0.0492	0.3592	0.3600	0.5697	0.5692
10, 5, 5, 5	0.0474	0.0474	0.0889	0.0896	0.1633	0.1624
20, 10, 10, 10	0.0495	0.0495	0.1536	0.1541	0.3175	0.3174
30, 20, 20, 20	0.0503	0.0509	0.3003	0.3002	0.5458	0.5473
5, 6, 7, 8	0.0515		0.0931		0.1817	
15, 16, 17, 18	0.0499		0.2233		0.4116	
25, 26, 27, 28	0.0472		0.3560		0.5869	
8, 7, 6, 5	0.0489		0.1093		0.1739	
18, 17, 16, 15	0.0538		0.2306		0.4288	
28, 27, 26, 25	0.0485		0.3609		0.6098	
5, 5, 10, 10	0.0490		0.0885		0.1803	
10, 10, 20, 20	0.0468		0.1631		0.3147	
15, 15, 30, 30	0.0530		0.2434		0.4482	
10, 10, 5, 5	0.0517		0.1047		0.1592	
20, 20, 10, 10	0.0511		0.1926		0.3288	
30, 30, 15, 15	0.0522		0.2781		0.4993	

**Table 2:** The estimated type I error rates and powers for *k* = 5.

	$\left(\sigma_{0}^{2},\sigma_{1}^{2},\;\sigma_{2}^{2},\;\sigma_{3}^{2},\sigma_{4}^{2},\sigma_{5}^{2}\right)$					
	(2, 2, 2, 2, 2, 2)		(2, 2, 2, 2, 1, 1)		(2, 2, 1, 1, 1, 1)	
(*n*_0_, *n*_1_,*n*_2_, *n*_3_, *n*_4_, *n*_5_)	MIM	Spurrier	MIM	Spurrier	MIM	Spurrier
5, 5, 5, 5, 5, 5	0.0495	0.0493	0.0960	0.0965	0.1351	0.1352
15, 15, 15, 15, 15, 15	0.0494	0.0499	0.2565	0.2567	0.3739	0.3733
30, 30, 30, 30, 30, 30	0.0496	0.0496	0.4786	0.4790	0.6269	0.6266
5, 10, 10, 10, 10, 10	0.0515	0.0510	0.1403	0.1401	0.2107	0.2099
10, 20, 20, 20, 20, 20	0.0517	0.0519	0.2588	0.2590	0.3460	0.3480
20, 30, 30, 30, 30, 30	0.0514	0.0514	0.4194	0.4194	0.5445	0.5446
10, 5, 5, 5, 5, 5	0.0482	0.0482	0.0990	0.0983	0.1442	0.1442
20, 10, 10, 10, 10, 10	0.0493	0.0494	0.1876	0.1868	0.2912	0.2912
30, 20, 20, 20, 20, 20	0.0493	0.0496	0.3674	0.3665	0.5396	0.5390
5, 6, 7, 8, 9, 10	0.0526		0.0994		0.1672	
15, 16, 17, 18, 19, 20	0.0522		0.2615		0.3930	
25, 26, 27, 28, 29, 30	0.0520		0.4260		0.5749	
10, 9, 8, 7, 6, 5	0.0493		0.1012		0.1769	
20, 19, 18, 17, 16, 15	0.0491		0.3101		0.4286	
30, 29, 28, 27, 26, 25	0.0498		0.4617		0.6111	
5, 5, 5, 10, 10, 10	0.0495		0.0803		0.1250	
10, 10, 10, 20, 20, 20	0.0496		0.1578		0.2515	
15, 15, 15, 30, 30, 30	0.0486		0.2495		0.3812	
10, 10, 10, 5, 5, 5	0.0487		0.1233		0.1548	
20, 20, 20, 10, 10, 10	0.0500		0.2355		0.3356	
30, 30, 30, 15, 15, 15	0.0518		0.3589		0.4853	

**Table 3:** The estimated type I error rates and powers for *k* = 7.

	$\left(\sigma_{0}^{2},\sigma_{1}^{2},\;\sigma_{2}^{2},\;\sigma_{3}^{2},\sigma_{4}^{2},\sigma_{5}^{2},\sigma_{6}^{2},\sigma_{7}^{2}\right)$					
	(2, 2, 2, 2, 2, 2, 2, 2)		(2, 2, 2, 2, 2, 2, 1, 1)		(2, 2, 2, 1, 1, 1, 1 1)	
(*n*_0_, *n*_1_, *n*_2_, *n*_3_, *n*_4_, *n*_5_, *n*_6_, *n*_7_)	MIM	Spurrier	MIM	Spurrier	MIM	Spurrier
5, 5, 5, 5, 5, 5, 5, 5	0.0528	0.0525	0.0853	0.0854	0.1273	0.1271
15, 15, 15, 15, 15, 15, 15, 15	0.0518	0.0519	0.2032	0.2025	0.3561	0.3573
30, 30, 30, 30, 30, 30, 30, 30	0.0456	0.0454	0.4265	0.4254	0.6225	0.6228
5, 10, 10, 10, 10, 10, 10, 10	0.0495	0.0494	0.1225	0.1221	0.1949	0.1954
10, 20, 20, 20, 20, 20, 20, 20	0.0492	0.0493	0.2245	0.2244	0.3379	0.3392
20, 30, 30, 30, 30, 30, 30, 30	0.0495	0.0496	0.3632	0.3626	0.5425	0.5412
10, 5, 5, 5, 5, 5, 5, 5	0.0505	0.0500	0.0861	0.0857	0.1277	0.1279
20, 10, 10, 10, 10, 10, 10, 10	0.0546	0.0544	0.1597	0.1597	0.2819	0.2821
30, 20, 20, 20, 20, 20, 20, 20	0.0507	0.0509	0.3222	0.3232	0.5239	0.5260
5, 6, 7, 8, 9, 10, 11, 12	0.0506		0.0868		0.1483	
15, 16, 17, 18, 19, 20, 21, 22	0.0509		0.2340		0.3747	
25, 26, 27, 28, 29, 30, 31, 32	0.0475		0.3904		0.5754	
12, 11, 10, 9, 8, 7, 6, 5	0.0494		0.1007		0.1846	
22, 21, 20, 19, 18, 17, 16, 15	0.0545		0.2811		0.4499	
32, 31, 30, 29, 28, 27, 26, 25	0.0484		0.4327		0.6384	
5, 5, 5, 5, 10, 10, 10, 10	0.0504		0.0697		0.1119	
10, 10, 10, 10, 20, 20, 20, 20	0.0509		0.1276		0.2240	
15, 15, 15, 15, 30, 30, 30, 30	0.0518		0.2011		0.3515	
10, 10, 10, 10, 5, 5, 5, 5	0.0508		0.1018		0.1550	
20, 20, 20, 20, 10, 10, 10, 10	0.0523		0.2122		0.3357	
30, 30, 30, 30, 15, 15, 15, 15	0.0541		0.3175		0.5011	

When the sample sizes (*n*_0_, *n*_1_, …, *n*_*k*_) are equal, it is easy to see that both the MIM test and the Spurrier test can exactly control the type I error rate. Moreover, the powers and the Type I error rates of the MIM test and Spurrier test are almost the same. One possible reason is that both tests utilize the same information from the given data. Specifically, they calculate the minimum sample variance among the treatment groups and the sample variance within the control group. Hence, the *p* value of Spurrier’s test (4) and plausibility of the MIM test (29) are similar in expression. Moreover, the pivots statistics of Spurrier’s test are ordered statistics of the multivariate *F* distribution. Although IM theory is different from classical statistics, we may conclude that the distribution function F in (29) is connected to the ordered statistics of the multivariate *F* distribution. However, Spurrier’s test does not work when the sample sizes of the treatment groups are unequal. In these cases, the proposed MIM test also has a type I error rate at a preset level, *α* ∈ (0.0457, 0.0543). Therefore, the MIM test outperforms Spurrier’s test because it has more flexible applicability while maintaining competitive efficiency.

**Fig 1:**
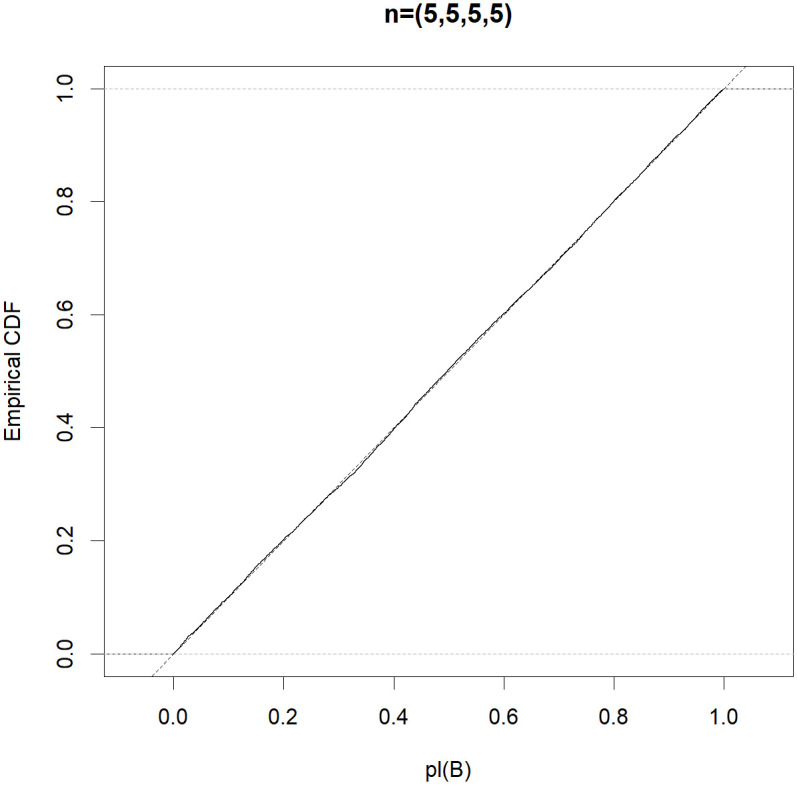
Empirical distribution functions of *pl(B)* (solid) compared with that of *Unif(0*,*1)* (dotted) based on the random samples, where (*n*_0_, *n*_1_, *n*_2_, *n*_3_) = (5, 5, 5, 5).

**Fig 2:**
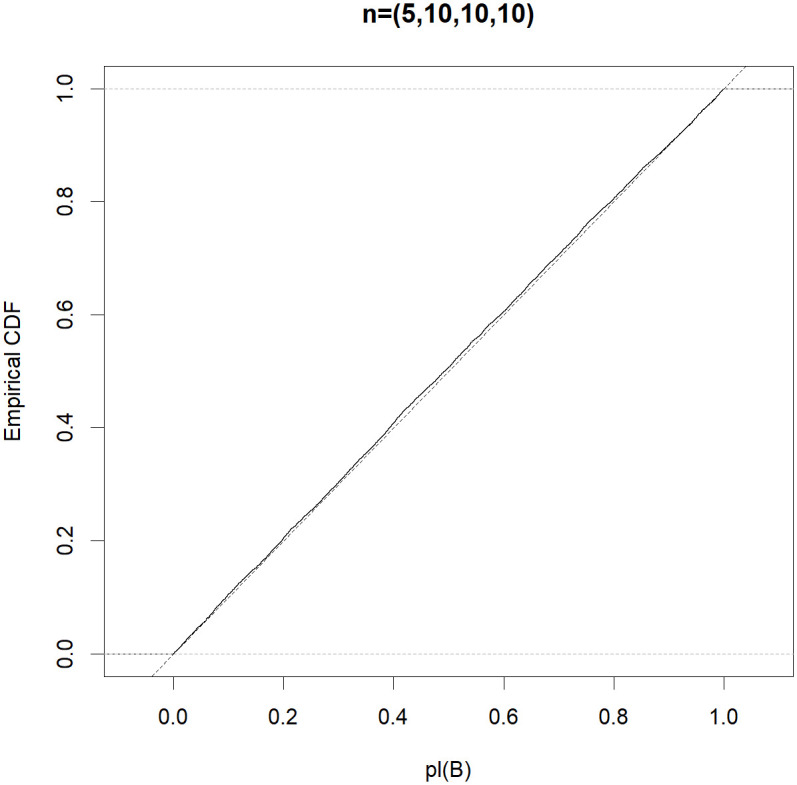
Empirical distribution functions of *pl(B)* (solid) compared with that of *Unif(0*,*1)* (dotted) based on the random samples, where (*n*_0_, *n*_1_, *n*_2_, *n*_3_) = (5, 10, 10, 10).

**Fig 3:**
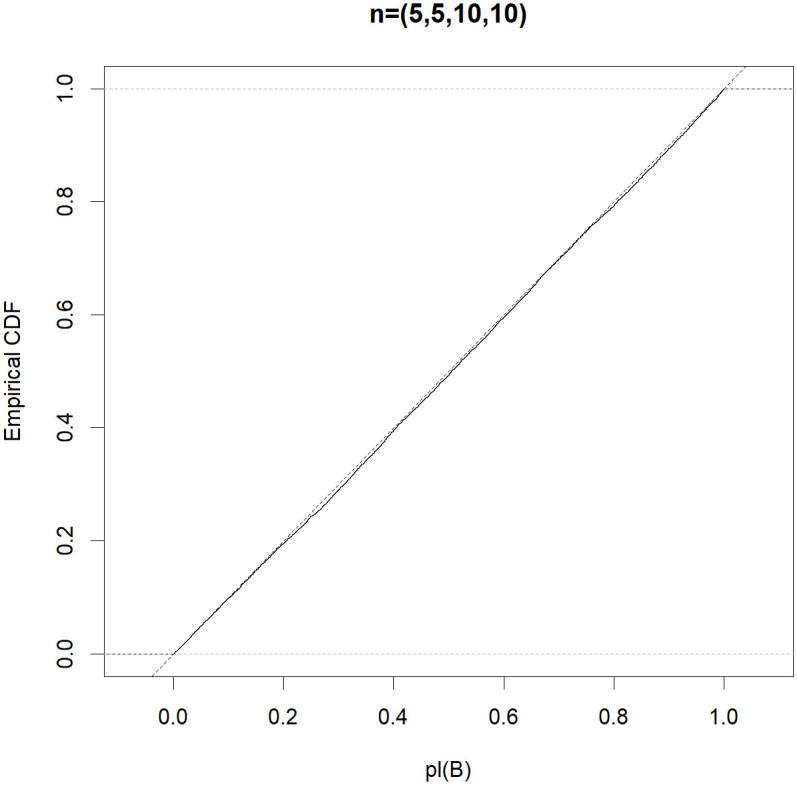
Empirical distribution functions of *pl(B)* (solid) compared with that of *Unif(0*,*1)* (dotted) based on the random samples, where (*n*_0_, *n*_1_, *n*_2_, *n*_3_) = (5, 5, 10, 10).

**Fig 4:**
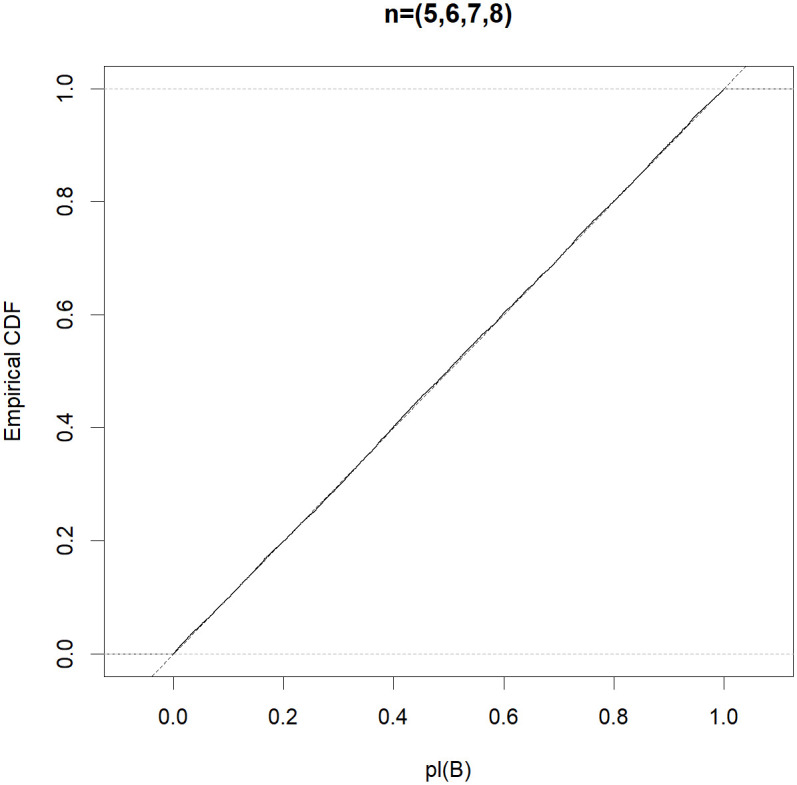
Empirical distribution functions of *pl(B)* (solid) compared with that of *Unif(0*,*1)* (dotted) based on the random samples, where (*n*_0_, *n*_1_, *n*_2_, *n*_3_) = (5, 6, 7, 8).

To be more informative, the efficient property in Theorem 1 is automatic if the Monte Carlo approximation *pl*(*B*) follows the uniform distribution in (0,1). To obtain a better understanding of the good performance of the MIM test, for each (*n*_0_, *n*_1_, *n*_2_, *n*_3_) ∈ {(5, 5, 5, 5), (5, 10, 10, 10), (5, 5, 10, 10), (5, 6, 7, 8)}, letting *μ*_1_ = *μ*_2_ = 0 and $\;\sigma_{0}^{2}=\sigma_{1}^{2}=\sigma_{2}^{2}=\sigma_{3}^{2}=1$, we generate 10,000 normal random samples and obtain a Monte Carlo estimate of the distribution function of *pl*(*B*). Figs 1–4 show that the distribution function of the approximate *pl*(*B*) is sufficiently close to that of *Unif(0*,*1)*. Therefore, the MIM test controls the type I error rate exactly.

## Applications

In this section, we use two real examples to illustrate the proposed MIM test.

***Example 1*** [2] considered four groups of men in the 35–45 years age bracket: (i) nonsmokers (control), (ii) former smokers (treatment), (iii) light smokers (treatment), and (iv) heavy smokers (treatment). Each group consisted of ten men and Table 4 shows the testosterone levels measured in *μ*g/dl. It is known that smoking has a negative impact on testosterone levels, leading to less variability, as some of them may have low testosterone levels for other reasons, while healthy people will have high levels. One question then asked is whether any of the testosterone levels among the three groups of smokers (including former smokers, light smokers and heavy smokers) have less variability than nonsmokers.

**Table 4:** Testosterone levels of men in the four groups.

Group	Testosterone level (μg/dl)	Mean	Variance
Non-smokers	0.32, 0.43, 0.99, 0.95, 0.92, 0.56, 0.87, 0.64, 0.78, 0.72	0.72	0.0520
Former smokers	0.36, 0.93, 0.40, 0.86, 0.85, 0.51, 0.76, 0.58, 0.73, 0.65	0.66	0.0389
Light smokers	0.82, 0.37, 0.77, 0.42, 0.74, 0.44, 0.48, 0.51, 0.61, 0.60	0.58	0.0250
Heavy smokers	0.29, 0.53, 0.33, 0.34, 0.52, 0.50, 0.49, 0.47, 0.40, 0.45	0.43	0.0075

By calculation, the *p*-values of the four groups by the Shapiro–Wilk normality tests are 0.5540, 0.6516, 0.4525 and 0.2398, respectively, and we accept the normality assumption for the data. These four groups give sample variances of 0.0520, 0.0389, 0.0250 and 0.0075. Moreover, the MIM and Spurrier tests have the same *p*-value = 0.0117. Hence, we can reject the null hypothesis at significance level *α* = 0.05, i.e., at least one smoking group has less variability in testosterone levels than the nonsmoking group.

**Fig 5:**
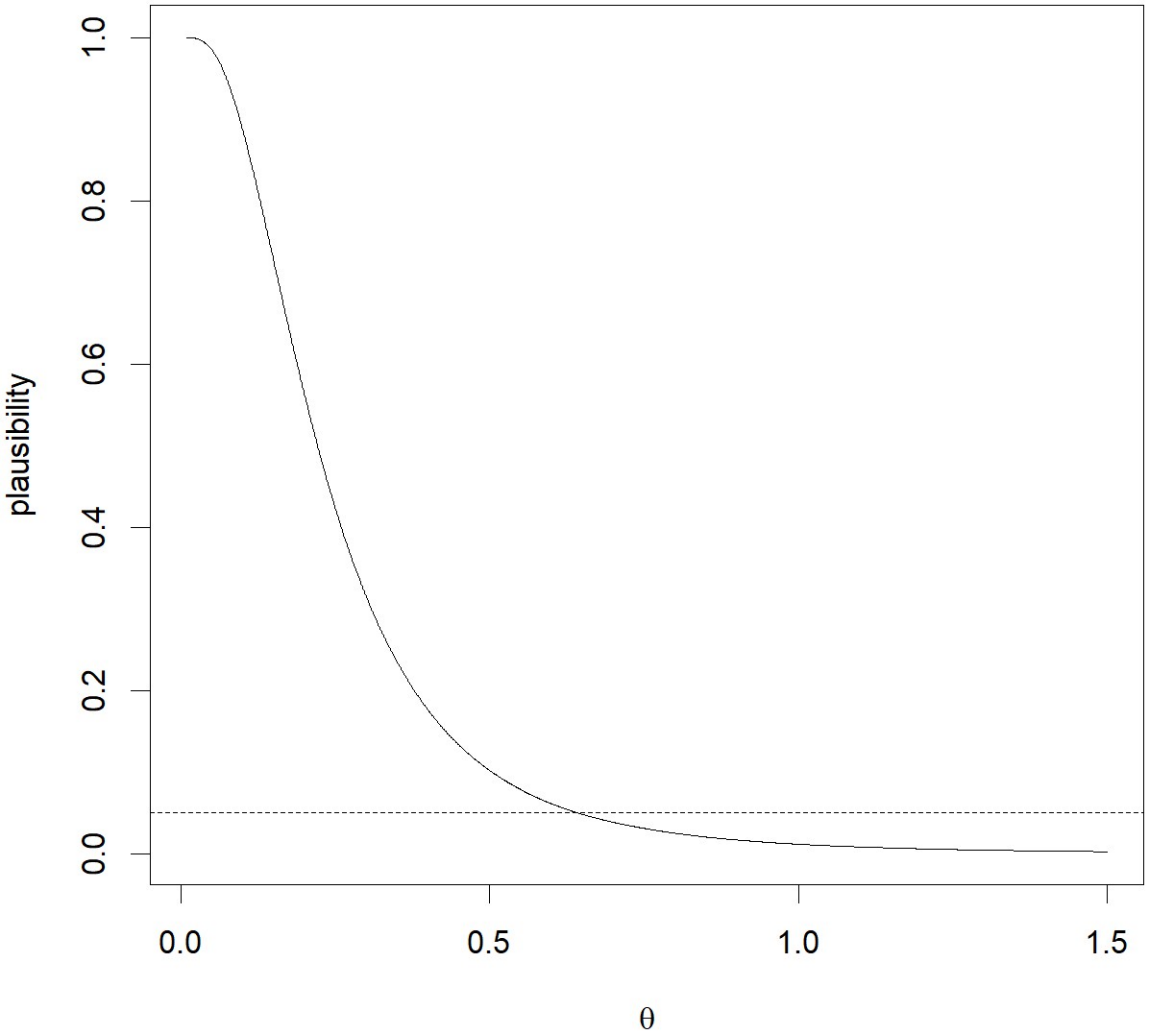
Plausibility function of the MIM based on the sample variance (*S*02, *S*12, *S*22, *S*32) = (0.0520, 0.3893, 0.2496, 0.0075), as a function of *θ*.

Different from other methods, the MIM method has a significant advantage in that it can provide probabilistic summaries of the information in data concerning the quantity of interest *B*. To be more informative, we plot the plausibility function *pl*(*B*), where *B* = {*θ*}, as a function of *θ* in Fig 5. By locating *α* on the vertical axis, the corresponding 1 − *α* MIM confidence interval can be easily obtained. More importantly, each point in the MIM interval is individually sufficiently plausible.

***Example 2*** To demonstrate the flexibility of the MIM method, the second dataset shown in Table 5 [3] contains blood count measurements on three groups of animals, one of which served as a control while the other two were treated with two drugs. Since the Shapiro–Wilk normality tests of the three groups give *p*-values of 0.4834, 0.6942 and 0.5483, we accept the normality assumption for this dataset. Moreover, the sample variances of the control, Drug A and Drug B groups are 0.8841, 0.8165 and 2.4240, respectively.

**Table 5:** Blood counts in the three groups.

Group	Blood Counts (millions of cells per cubic millimeter)	Mean	Variance
Control	7.40, 8.50, 7.20, 8.24, 9.84, 8.32	8.25	0.8841
Drug A	9.76, 8.80, 7.68, 9.36	8.90	0.8165
Drug B	12.80, 9.68, 12.16, 9.20, 10.55	10.88	2.4240

**Fig 6:**
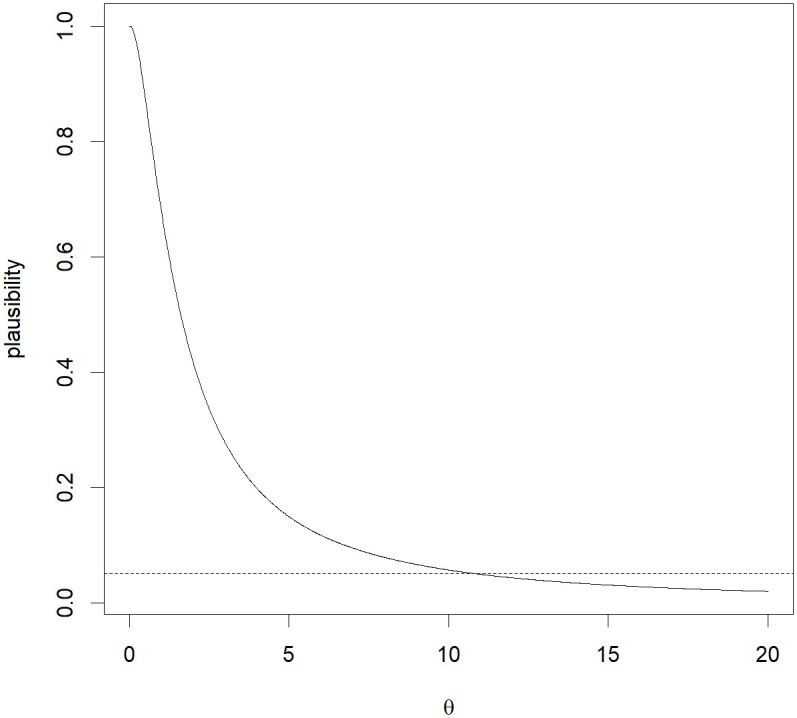
Plausibility function of the MIM based on the sample variance (*S*02, *S*12, *S*22) = (0.8841, 0.8165, 2.4240), as a function of *θ*.

The problem of interest is to test whether the variability in blood counts following treatment with Drug A and Drug B is smaller than that in the control. Due to accidental losses, existing methods cannot be applied to these data because the numbers of animals in the three groups are unequal. As an alternative, from Fig 6, the plausibility function of the MIM gives a plausibility of 0.6804 in this situation. Hence, there is no significant evidence suggesting rejecting the null hypothesis that the variability in blood counts following treatment with Drug A and Drug B is the same as that of the control.

## Discussion

In applied statistics, there often exists a common and important problem comparing the variances of experimental treatments with that of a standard or control treatment under the assumption that the measurements are independent and normally distributed. The existing test procedures are not well developed for testing the homogeneity of variances under a control group and require the sample sizes to be equal. In real data analysis, the requirement for equal sample sizes of experimental treatments may not be satisfied because of accidental losses or other unexpected circumstances. To date, a more general test method is needed.

It is crucial to construct an appropriate nonprior, frequency calibrated testing method. In this paper, we propose a new test method based on the marginal inferential model framework. The proposed MIM method has at least three contributions as follows: first, different from the general IM framework, the new MIM method utilizes partial information from the null hypothesis to construct accurate testing methods. This idea complements the precise theory of statistical inference. Second, the constructed association model in (26) is the key to accurate inference of the MIM. Since the distribution of the righthand side of equation (25) does not rely on the observable data, we demonstrate that the MIM method has an accurate type I error rate but does not require simulation verification. Finally, the MIM test does not require the sample size among the treatment groups to be equal, while other tests require the sample size to be equal. Note that the MIM has an advantage in providing valid probabilistic uncertainty quantification. Unlike the p value of Spurrier’s test, the output of the MIM test, i.e., plausibility, is posterior-probabilistic in nature and therefore has a meaningful interpretation within and not just across experiments. Therefore, the plausibility function provides more information than the *p* value of the frequentist approach because even a large *p* value cannot “confirm” the truth of the null hypothesis.

As we focus on comparing the variances of the normal distribution, this implies that the underlying distribution of the data needs to be normally distributed. A potential challenge of the proposed procedure (as well as other methods based on parametric models) is that we do not know the accurate distribution of the data. In our real-data applications, we apply the Shapiro–Wilk normality test to test whether the data are normally distributed. Even though the p values in two real-data examples are greater than a specified significance level, the data are still likely to be nonnormal, especially with a relatively small sample size. One possible way to alleviate this challenge is to increase the sample size of the data.

Different from traditional IM-based test methods, the proposed MIM solution uses part of the information given in the null hypothesis to reduce the dimension of the auxiliary variable and gains more validity. This idea could be applied to other multiple comparison procedures for comparing several treatments with a control. For instance, the methodology can be extended to the comparison of the mean of the normal distribution, where the mean is of interest and the variance is a nuisance parameter. In this case, the marginal association (e.g., Eqs (17) and (24)–(26)) would be equations concerning the normal mean. Comparison for the mean is more complicated since different marginalization techniques might result in varying inferential results. Indeed, we are looking for the best marginalization technique for the comparison of the mean. Some studies are still ongoing. Finally, for two-sided tests, since the dimension of the auxiliary variable is two-dimensional, there could be interest in the simultaneous prediction of several auxiliary variables. The optimal predictive random set needs further study.

## Supporting information

S1 Appendix R codes for calculation. (DOCX)
