# Supplementary material for: Comparing the variances of several treatments with that of a control treatment: Theory and applications
Source: PLoS One. 2024 Jan 12;19(1):e0296376. doi: 10.1371/journal.pone.0296376 (PMC10786376; doi:10.1371/journal.pone.0296376)
Supplement: S1 Appendix — (DOCX) [file pone.0296376.s001.docx]

1. Type I error rate and power

parameter<-c(5,5,5,5,5) #sample sizes

Prfun<-function(x,c,m,n,p) (1-pchisq(x*c*(m-1)/(n-1),df=m-1))^p*dchisq(x,df=n-1)

k<-length(parameter)-1

n.vector<-parameter

sig.square<-c(1,1,1,1,1)

alpha=0.05

NN=10000

N=10000

pl.values<-c()

n.IM<-n.Spurrier<-0

V<-matrix(0,nrow = k+1,ncol = NN)

for (t in 1:(k+1)) {

V[t,]<-rchisq(NN,n.vector[t]-1)/(n.vector[t]-1)

}

V0<-V[1,]

V.min<-apply(V[2:(k+1),],2,min)

for (i in 1:N) {

S.square<-rep(0,k+1)

for (t in 1:(k+1)) {

S.square[t]<-var(rnorm(n.vector[t],0,sqrt(sig.square[t])))

}

## MIM pl-value

NS<-S.square

sig0.estimeted<-NS[1]/V0

sig.estimated<-min(NS[-1])/V.min

theta<-sig.estimated/sig0.estimeted

pl<-1-sum(theta<=1)/NN

if(pl<alpha){n.IM<-n.IM+1}

pl.values[i]<-pl

## Spurrier p-value

if(max(parameter[-1])-min(parameter[-1])==0){

c.hat<-min(S.square[-1])/S.square[1]

Prf<-function(x) Prfun(x,c=c.hat,m=parameter[2],n=parameter[1],p=k)

pvalue_of_Spurrier<-1-integrate(Prf,0,Inf)$value

if(pvalue_of_Spurrier<alpha){n.Spurrier<-n.Spurrier+1}

}else{

n.Spurrie<-0

}

}

r<-c(n.IM,n.Spurrier)/N ## Type I error or power

r

plot(ecdf(pl.values),xlab = "pl(B)",ylab="Empirical CDF",main="") ## Empirical CDF of plausibility function

abline(a=0,b=1,lty=2)

2. Example 1

x1<-c(0.32, 0.43, 0.99, 0.95, 0.92, 0.56, 0.87, 0.64, 0.78, 0.72)

x2<-c(0.36, 0.93, 0.40, 0.86, 0.85, 0.51, 0.76, 0.58, 0.73, 0.65)

x3<-c(0.82, 0.37, 0.77, 0.42, 0.74, 0.44, 0.48, 0.51, 0.61, 0.60)

x4<-c(0.29, 0.53, 0.33, 0.34, 0.52, 0.50, 0.49, 0.47, 0.40, 0.45)

shapiro.test(x4)

parameter<-c(length(x1),length(x2),length(x3),length(x4)) ##sample sizes

Prfun<-function(x,c,m,n,p) (1-pchisq(x*c*(m-1)/(n-1),df=m-1))^p*dchisq(x,df=n-1)

k<-length(parameter)-1

n.vector<-parameter

#sig.square<-c(2,1.75,1,5,1.25,1,0.75)

alpha=0.05

NN=1000000

V<-matrix(0,nrow = k+1,ncol = NN)

for (t in 1:(k+1)) {

V[t,]<-rchisq(NN,parameter[t]-1)/(n.vector[t]-1)

}

V0<-V[1,]

V.min<-apply(V[2:(k+1),],2,min)

## MIM pl-value

S.square<-c(var(x1),var(x2),var(x3),var(x4))

NS<-S.square

sig0.estimeted<-NS[1]/V0

sig.estimated<-min(NS[-1])/V.min

theta<-sig.estimated/sig0.estimeted

pl<-1-sum(theta<=1)/NN

## Spurrier p-vaue

c.hat<-min(S.square[-1])/S.square[1]

Prf<-function(x) Prfun(x,c=c.hat,m=parameter[2],n=parameter[1],p=k)

pvalue_of_Spurrier<-1-integrate(Prf,0,Inf)$value

print(c(pl,pvalue_of_Spurrier))

## plausibility function

pl_values<-c()

aa<-seq(0.01,1.5,0.01)

for (i in 1:length(aa)) {

pl_values[i]<-1-sum(theta<=aa[i])/NN

}

plot(x=aa,y=pl_values,xlab = expression(theta),ylab = "plausibility",type = "l")

abline(a=0.05,b=0,lty=2)

3. Example 2

x1<-c(7.40, 8.50, 7.20, 8.24, 9.84, 8.32)

x2<-c(9.76, 8.80, 7.68, 9.36)

x3<-c(12.80, 9.68, 12.16, 9.20, 10.55)

shapiro.test(x1)

parameter<-c(length(x1),length(x2),length(x3)) ##sample sizes

Prfun<-function(x,c,m,n,p) (1-pchisq(x*c*(m-1)/(n-1),df=m-1))^p*dchisq(x,df=n-1)

k<-length(parameter)-1

n.vector<-parameter

#sig.square<-c(2,1.75,1,5,1.25,1,0.75)

alpha=0.05

NN=1000000

V<-matrix(0,nrow = k+1,ncol = NN)

for (t in 1:(k+1)) {

V[t,]<-rchisq(NN,parameter[t]-1)/(n.vector[t]-1)

}

V0<-V[1,]

V.min<-apply(V[2:(k+1),],2,min)

## MIM pl-value

S.square<-c(var(x1),var(x2),var(x3))

NS<-S.square

sig0.estimeted<-NS[1]/V0

sig.estimated<-min(NS[-1])/V.min

theta<-sig.estimated/sig0.estimeted

pl<-1-sum(theta<=1)/NN

pl

## plausibility function

pl_values<-c()

aa<-seq(0.01,20,0.01)

for (i in 1:length(aa)) {

pl_values[i]<-1-sum(theta<=aa[i])/NN

}

plot(x=aa,y=pl_values,xlab = expression(theta),ylab = "plausibility",type = "l")

abline(a=0.05,b=0,lty=2)
